# (+)-*N*-(2-Hydroxypropyl)lindcarpine: A New Cytotoxic Aporphine Isolated from *Actinodaphne pruinosa* Nees

**DOI:** 10.3390/molecules14082850

**Published:** 2009-07-31

**Authors:** Tiah Rachmatiah, Mat Ropi Mukhtar, Mohd Azlan Nafiah, Muhammad Hanafi, Soleh Kosela, Hiroshi Morita, Marc Litaudon, Khalijah Awang, Hanita Omar, A. Hamid A. Hadi

**Affiliations:** 1 Department of Chemistry, Faculty of Science and Mathematic, University of Indonesia, Depok, Indonesia; E-mail: tiahrachmatiah@yahoo.com (T.R.); 2 Department of Pharmacy, Faculty of Science and Mathematic, National Institute of Science and Technology, Jakarta, Indonesia; 3 Centre for Natural Products and Drug Discovery, Block D, Department of Chemistry, Faculty of Science, University of Malaya, 50603 Kuala Lumpur, Malaysia; E-mails: matropi@um.edu.my (M.R.M.), khalijah@um.edu.my (K.A.); 4 Chemistry Department, Faculty of Science and Technology, University of Pendidikan Sultan Idris, Tg. Malim, Perak, Malaysia; E-mail: azlan@upsi.edu.my (M.A.N.); 5 Research Center for Chemistry, Indonesian Institute of Sciences, Bandung, Indonesia; 6 Faculty of Pharmaceutical Sciences, Hoshi University, Ebara 2-4-41 Shinagawa, Tokyo 142-8501, Japan; E-mail: moritah@hoshi.ac.jp (H.M.); 7 Institut de Chimie des Substances Naturelles, Centre Nationale de la Recherche Scientifique, UPR2301, 91198, Gif-sur-Yvette, Cedex, France; E-mail: marc.litaudon@icsn.cnrs-gif.fr (M.L.)

**Keywords:** *Actinodaphne pruinosa*, Lauraceae, aporphine alkaloid, cytotoxic

## Abstract

One new alkaloid; (+)-*N*-(2-hydroxypropyl)lindcarpine (**1**), together with four known aporphine alkaloids, (+)-boldine (**2**) (+)-norboldine (**3**), (+)-lindcarpine (**4**) and (+)-methyllindcarpine (**5**) were isolated from the stem bark of *Actinodaphne pruinosa* Nees (Lauraceae). (+)-*N*-(2-Hydroxypropyl)lindcarpine (**1**) exhibited cytotoxic activity against P-388 murine leukemia cells with an IC_50_ value of 3.9 μg/mL. Structural elucidation of all the compounds were performed by spectral methods such as 1D- and 2D- NMR, IR, UV, and HRESIMS.

## Introduction

*Actinodaphne pruinosa* is a tree of moderate size (about 30-40 feet) found in Peninsular Malaysia and Jawa, Indonesia. Locally, *Actinodapne* is known as *wuru* (Indonesia) or *medang kuning* and *medang kunyit* (Malaysia) [1,2]. *Actinodaphne* plants of the family Lauraceae have been reported to produce isoquinoline alkaloids (aporphines, oxoaporphines) and lactones [3,4]. These alkaloids are of some pharmacological importance, as exemplified by liriodenine, an oxoaporphine, which was reported to have antitumor, antibacterial, and antifungal activities [5]. In addition, dicentrine, an aporphine, was known to have cytotoxic activity against P-388 murine cells [6]. In the present paper, the isolation and characterization of new aporphine; (+)-*N*-(2-hydroxypropyl)lindcarpine (**1**) is described. This alkaloid, together with four known alkaloids, (+)-boldine (**2**) [7], (+)-norboldine (**3**) [7], (+)-lindcarpine (**4**) [7,8] and (+)-methyllindcarpine (**5**) [8], were obtained from a CH_2_Cl_2_ extract of the stem bark of *Actinodaphne pruinosa.*

**Figure 1:**
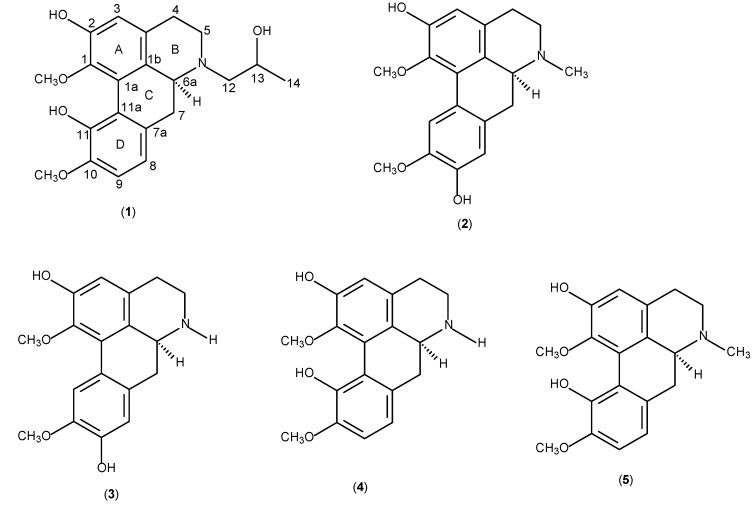
Alkaloids **1- 5** isolated from *Actinodaphne pruinosa*.

## Results and Discussion

(+)-*N*-(2-Hydroxypropyl)lindcarpine (**1**; see Figure 1) exhibited a molecular formula of C_21_H_26_NO_5 _based on the HRESIMS spectrum (positive mode) which showed a pseudomolecular ion at *m/z* 372.1797 [M+H]^+^ (calcd. 372.1811, Δ-1.4 mmu). The IR spectrum revealed an absorption band at 3,180 cm^-1^ due to the OH stretching vibration. The overall physical properties and NMR spectral profile revealed its identity as a member of the aporphine group of isoquinoline, a characteristic and distinguishable chemical marker of *Actinodaphne* plants [3,4].

In the ^1^H-NMR spectrum (Table 1) the presence of a methyl group attached to -CH(OH)- signal at δ 1.22 (3H, *d*, *J* = 6.1 Hz); two methoxyl signals at δ 3.65 and δ 3.92; three aromatic protons at δ 6.79 (*s*, H-3), δ 6.84 (*d*, 8.0 Hz, H-8) and δ 6.86 (*d*, 8.0 Hz, H-9) were observed. The ^13^C-NMR (Table 1) and DEPT spectra, showed a total of 21 carbon signals; three methyls, four methylenes, one methine bearing hydroxyl group, four methines, and nine quaternary carbons in which four are aromatic oxygenated carbon signals. 

**Table 1:** ^1^H-NMR (400 MHz) and ^13^C-NMR (100 MHz) spectral data of compound **1** in CDCl_3_ (*δ* in ppm, *J* in Hz).

Position	δ^ 1^H (Hz)	δ^ 13^C	HMBC (*^2^J, ^3^J*)
1		140.6	
1a		125.0	
1b		131.2	
2		147.6	
3	6.79 *s*	114.0	1, 2, 3a, 4
3a		129.2	
4	2.67 *m*	29.1	
	2.71 *m*		1b
5	3.08 *m*	52.3	12, 6a
6a	3.30 *dd* (13.1, 3.2)	61.9	12
7	2.90 *dd* (13.1, 3.2)	36.7	6a
	2.56 *t* (13.1)		
7a		129.9	
8	6.84 *d* (8.0)	119.5	7, 11a, 10
9	6.86 *d* (8.0	111.4	7a, 11
10		149.2	
11		143.2	
11a		119.6	
12	2.80 *m*	63.3	5, 6a
	2.38 *dd* (13.7, 9.0)		5, 13
13	3.89 *m*	66.1	
14 (Me)	1.22 *d* (6.1)	20.8	13
1-OMe	3.65 *s*	62.4	1
10-OMe	3.92 *s*	56.4	10

**Figure 2:**
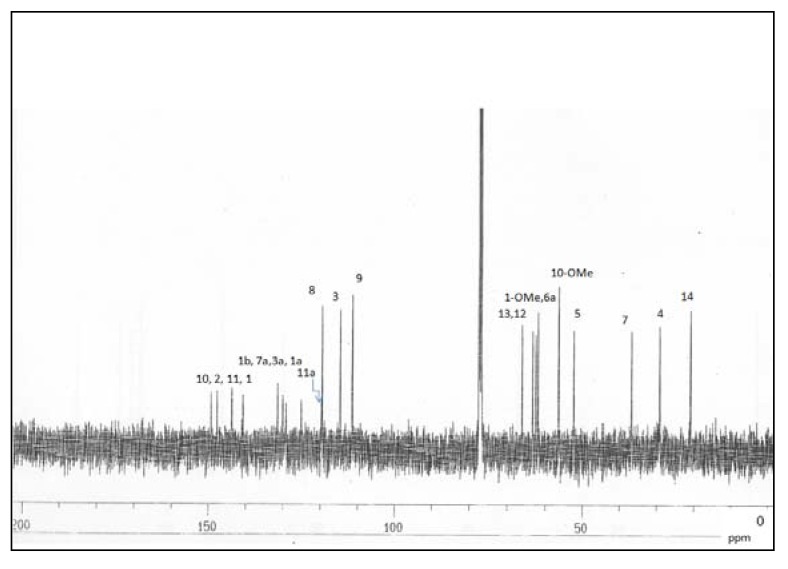
^13^C-NMR spectrum of alkaloid **1**.

**Figure 3:**
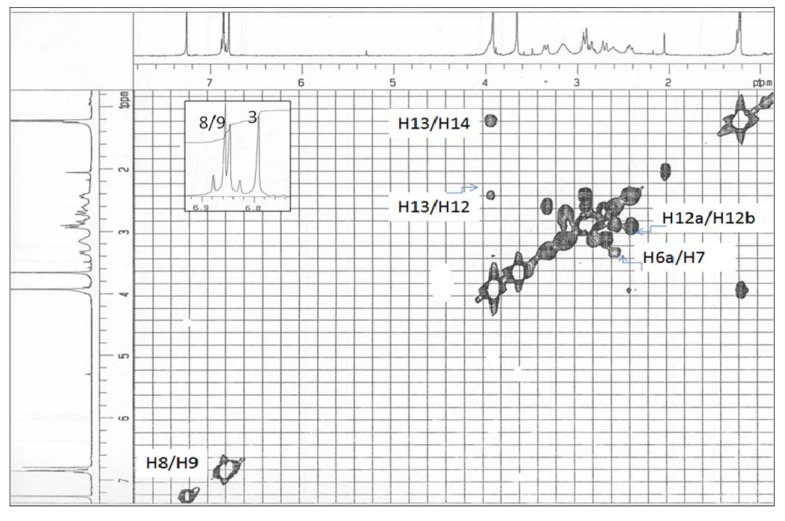
COSY spectrum of alkaloid **1**.

**Figure 4:**
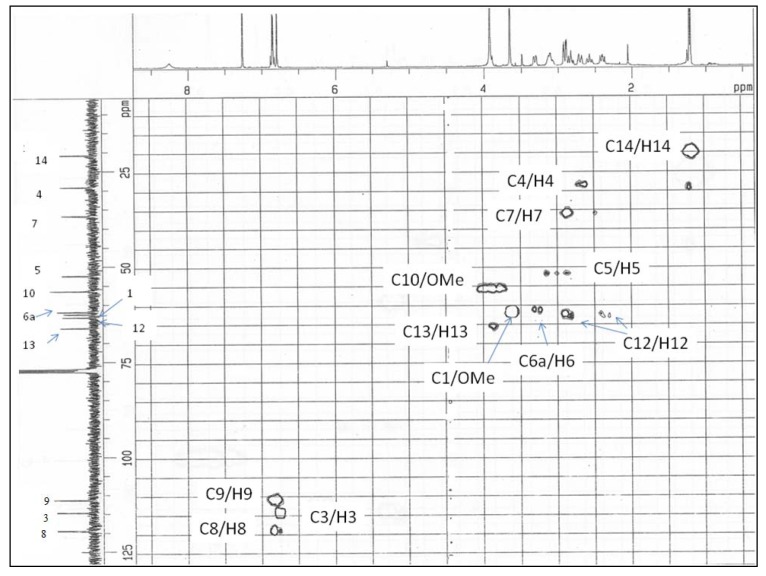
HMQC spectrum of alkaloid **1**.

The complete ^1^H- and ^13^C-NMR (Figure 2) spectral assignment of **1** was accomplished by thorough analysis of DEPT, COSY (Figure 3), HMQC (Figure 4), and HMBC data. The ^1^H-^1^H COSY, combined with the HMQC spectrum revealed that **1 **has the following partial structure: - CH_2_CH_2_ - (C4 and C5); -CHCH_2_- (C6a-C7); =CHCH= (C8-C9); -CH_2_CH- (C12-C13); and –CHCH_3_- (C13-C14). All of these segments were compatible for rings B, C, and D of a 1,2,9,10-tetrasubstituted aporphine type linked to –CH_2_CH(OH)CH_3_ unit. The HMBC spectrum of **1** provided conclusive evidence for the presence of the 2-hydroxypropyl chain unit. The HMBC spectrum showed cross peaks of H-3 with C1, C2, C3a and C4; H-12 with C5, C6a and C14; H-8 with C7, C11a and C-10; and H-9 with the C7a and C11.

The NOE differential measurements, showed enhancement of H-4 (δ 2.67) upon radiation of H-3 (δ 6.79). In addition the irradiation of H-9 showed enhancement of 10-OMe protons and H-8, suggesting that the methoxyl groups are placed at C-1 and C-10, respectively. The absolute configuration of the asymmetric carbon at C-13 was not determined due to the limited amount of compound available, so alkaloid **1** can be considered a racemic mixture.

To our knowledge, there has been no report on the phytochemical study and medicinal value of *Actinodaphne pruinosa*. This is the first report on the occurrence of *N*-(2-hydroxypropyl)aporphine type of alkaloid; (+)-*N*-(2-hydroxypropyl)lindcarpine (**1**) which exhibited significant cytotoxicity against P-388 murine leukemia cells. 

## Conclusions

In summary, we have observed that *Actinodaphne pruinosa* produces alkaloids closely related to those found in *A. nitida, A. acutivena, A. abovata and A. sesquipedalis* which were studied previously [4,9,10]. These species yielded noraporphine or *N*-methylaporphine types. The presence of *N*-(2-hydroxypropyl)aporphine type which significantly exhibited a potent cytotoxicity against P-388 [11] murine leukemia cells with IC_50_ 3.9 μg/mL, suggesting its potential for further investigation as anti-cancer agent. 

## Experimental

### General

The optical rotations were recorded on s Jasco (Japan) P1010 instrument equipped with a tungsten lamp. HRESIMS was obtained on a Thermo Finnigan Automass Multi. The ultraviolet spectra were obtained in MeOH on a Shimadzu UV-160A ultraviolet-visible spectrometer. The infrared spectra were recorded on a Perkin Elmer 1600 Double-Beam recording spectrometer, using chloroform as solvent. The ^1^H-NMR and ^13^C-NMR spectra were recorded in deuterated chloroform on a JEOL 400 MHz. Chemical shifts are reported in ppm on δ scale, and the coupling constants are given in Hz. Silica gel 60, 70-230 mesh ASTM (Merck 7734) and silica gel 60, 230-400 Mesh ASTM (Merck 9385) were used for column and flash chromatography, respectively. Mayer’s reagent was used for alkaloid screening. 

### Plant material

Stem bark of *Actinodaphne pruinosa*, collected at Bukit Bauk, Dungun, Terengganu, Malaysia, in May 2004 was identified by Mr. Teo Leong Eng. A voucher specimen (KL 5055) was deposited in the Herbarium of Department of Chemistry, University of Malaya, Malaysia and at the Herbarium of the Forest Research Institute, Kepong, Malaysia.

### Extraction and isolation of the alkaloids

The dried stem bark of *Actinodaphne pruinosa* (2.0 kg) was ground and extracted exhaustively for 12 hours by Soxhlet extraction with hexane, followed by CH_2_Cl_2_. Extraction of alkaloids was carried out in the usual manner, which has been described in detail [12,13] and gave 43.0 g of crude alkaloid. The crude alkaloid was submitted to exhaustive column chromatography over silica gel using CH_2_Cl_2_ gradually enriched with methanol to yield 26 fractions. Fractions were combined on the basis of TLC behavior. Fractions 24-25 (3.0 g), afforded three alkaloids identified as (+)-*N*-(2-hydroxypropyl)-lindcarpine (**1**) (0.21%, PTLC; CH_2_Cl_2_-MeOH 98:2), (+)-lindcarpine (**4**) (1.51%, PTLC; CH_2_Cl_2_-MeOH 95:5), (+)-methyllindcarpine (**5**) (1.89%, PTLC; CH_2_Cl_2_-MeOH 97:3). Fraction 19-20 (1.3 g) produced alkaloid **2**, identified as (+)-boldine (1.58%, PTLC; CH_2_Cl_2_-MeOH 95:5) [6]. (+)-Norboldine (**3**, 1.89%), was also separated by preparative TLC of fraction 26 (190 mg) over silica gel using CH_2_Cl_2_-MeOH; 95:5: saturated with NH_4_OH). 

(+)-*N-(2-Hydroxypropyl)lindcarpine***** (**1**, Figure 1): A colorless powder , [α]^25^_D_= + 120° (*c* = 0.02, MeOH), UV: λ_methanol_: 309 nm; IR ν_max_ (KBr): 3,180, 3,014, 2,934, 2,360, 1,594 cm^-1^; HRESIMS (positive mode): *m/z*: 372.1797 [M+H]^+^ (Calcd. 372.1811, Δ-1.4 mmu for C_21_H_26_NO_5_);^ 1^H-NMR (400 MHz, CDCl_3_) and ^13^C-NMR (100 MHz, CDCl_3_) see Table 1; Principle NOE’s (%) in CDCl_3_: OCH_3_-10 to H-9 (2.6%).

### Cytotoxic assays

The cytotoxicity of (+)-*N*-(2-hydroxypropyl)lindcarpine (**1**) against P-388 murine leukemia cells was tested using MTT-microculture tetrazolim assay [11].
